# Supplementary material for: Longitudinal Associations between the Neighborhood Built Environment and Cognition in US Older Adults: The Multi-Ethnic Study of Atherosclerosis
Source: Int J Environ Res Public Health. 2021 Jul 28;18(15):7973. doi: 10.3390/ijerph18157973 (PMC8345405; doi:10.3390/ijerph18157973)
Supplement: Supplementary file 1 [file ijerph-18-07973-s001.zip › Supplement_Tables_Figs_April17.pdf]

Table S1. Neighborhood characteristics by site

	Mean (SD)					
	Forsyth County, North Carolina	New York, New York	Baltimore, Maryland	St. Paul, Minnesota	Chicago, Illinois	Los Angeles, California
At Exam 5						
Social destin. density[a]	14.5 (15.1)	421.6 (275.4)	64.5 (69.5)	31.4 (29.0)	255.0 (299.1)	61.7 (53.6)
Walking destin. density[a]	4.9 (7.8)	240.6 (116.6)	20.7 (31.1)	14.5 (13.7)	89.8 (101.8)	31.2 (31.7)
Network ratio[a]	0.24 (0.12)	0.57 (0.13)	0.38 (0.19)	0.45 (0.17)	0.52 (0.14)	0.44 (0.14)
Proportion retail[a]	0.016 (0.033)	0.097 (0.036)	0.0124 (0.030)	0.011 (0.014)	0.084 (0.052)	0.050 (0.043)
Neighborhood SES[b]	-0.09 (0.93)	-1.08 (1.38)	-0.36 (0.88)	-0.04 (0.63)	-1.62 (1.29)	-0.12 (1.10)
Population density[a]	691 (394)	25,859 (10,028)	2,432 (1,793)	1,982 (1,014)	7,097 (5,034)	3,695 (2,247)
Average annual change (Exam 1 to 5)						
Social destin. density[a]	0.32 (1.51)	11.07 (16.57)	0.91 (4.11)	-0.64 (3.07)	5.23 (16.57)	1.02 (4.80)
Walking destin. density[a]	0.06 (0.78)	0.32 (9.05)	-0.44 (2.28)	-0.27 (2.10)	-1.41 (6.92)	-0.37 (2.73)
Network ratio[a]	-0.002 (0.010)	0.002 (0.009)	-0.003 (0.014)	-0.004 (0.015)	0.001 (0.011)	-0.001 (0.013)
Proportion retail[a]	-0.0001 (0.0029)	0.0012 (0.0024)	-0.0085 (0.0114)	-0.0039 (0.0071)	-0.0002 (0.0042)	-0.0002 (0.0043)

Abbreviations: destin = destination; SES = socioeconomic status; SD = standard deviation

[a] Measured in ½-mile radial buffer surrounding residence

[b] Measured at US Census tract level

Table S2. Changes in built environment by residential moves

Built environment changes	n (%)			
	Never moved n=1269	1 move n=369	≥2 moves n=178	Chi-square p-value
Social destination density				
<1SD from mean	0 (0.0%)	43 (11.7%)	17 (9.6%)	<.0001
±1SD from mean	1120 (88.3%)	302 (81.8%)	148 (83.2%)	
>1SD from mean	149 (11.7%)	24 (6.5%)	13 (7.3%)	
Walking destination density				
<1SD from mean	11 (0.9%)	49 (13.3%)	22 (12.4%)	<.0001
±1SD from mean	1206 (95.0%)	294 (79.7%)	142 (79.8%)	
>1SD from mean	52 (4.1%)	26 (7.1%)	14 (7.9%)	
Network ratio				
<1SD from mean	15 (1.2%)	123 (33.3%)	60 (33.7%)	<.0001
±1SD from mean	1201 (94.6%)	194 (52.6%)	88 (49.4%)	
>1SD from mean	53 (4.2%)	52 (14.1%)	30 (16.9%)	
Proportion retail				
<1SD from mean	113 (9.0%)	37 (10.1%)	23 (12.9%)	<.0001
±1SD from mean	1134 (90.1%)	303 (82.8%)	131 (73.6%)	
>1SD from mean	12 (1.0%)	26 (7.1%)	24 (13.5%)	

Table S3. Unadjusted association between built environment and maintained/improved cognition

	Maintained/improved CASI score[c]		Maintained/improved DSC score[c]	
At Exam 5[a,b]	OR (95% CI)	p-value	OR (95% CI)	p-value
Social destination density	1.00006 (0.99995, 1.00017)	0.31	1.00004 (0.99994, 1.00014)	0.45
Walking destination density	1.00009 (0.99985, 1.00032)	0.47	0.99996 (0.99974, 1.00018)	0.72
Network ratio	1.01 (0.89, 1.16)	0.83	0.86 (0.76,0.97)	0.01
Proportion retail	1.55 (0.95, 2.55)	0.08	1.16 (0.74,1.80)	0.52

Abbreviation: OR = Odds ratio; CI =Confidence Interval; CASI = Cognitive Abilities Screening Instrument; DSC = Digit Symbol Coding

[a] Measured in ½-mile radial buffer surrounding residence

[b] Continuous measures

[c] Maintained/improved score versus decline in score from Exam 5 to 6

Table S4. Unadjusted association between average annual built environment changes and maintained/improved cognition

	Maintained/improved CASI score[d]		Maintained/improved DSC score[d]	
Average annual change (Exam 1 to 5)[a,b]	OR (95% CI)	p-value	OR (95% CI)	p-value
Social destination density	1.002 (0.999, 1.004)	0.15	1.001 (0.999, 1.003)	0.35
Walking destination density	1.002 (0.997, 1.006)	0.51	1.001 (0.997, 1.006)	0.59
Network ratio	1.69 (0.27, 10.62)	0.58	0.73 (0.14, 3.98)	0.72
Proportion retail[c]	6.27 (0.08, 508.41)	0.41	0.20 (0.00, 10.54)	0.42

Abbreviation: OR = odds ratio; CI =Confidence Interval; CASI = Cognitive Abilities Screening Instrument; DSC = Digit Symbol Coding

[a] Continuous measures

[b] Measured in ½-mile radial buffer surrounding residence

[c] Variable was log transformed and top and bottom 1 percentile deleted to improve efficiency of the estimate

[d] Maintained/improved score versus decline in score from Exam 5 to 6

Table S5. Adjusted association between built environment and maintained/improved cognition, using Inverse Probability Weighted to account for attrition and selection bias

Built environment characteristic at Exam 5[a]	Buffer size	Maintained/improved CASI score[b,c]		Maintained/improved DSC score[b,c]	
		OR (95% CI)	p-value	OR (95% CI)	p-value
Social destination density (per 100)	½-mile	1.00 (0.99,1.01)	0.90	1.01 (1.00, 1.01)	0.14
Walking destination density (per 100)	½-mile	1.00 (0.98, 1.02)	0.81	1.02 (1.00, 1.03)	0.07
Network ratio	½-mile	0.98 (0.82, 1.16)	0.78	0.95 (0.82, 1.11)	0.53
Proportion retail	½-mile	1.20 (0.59, 2.43)	0.61	1.86 (0.99, 3.50)	0.06
Social destination density (per 100)	1-mile	1.00 (0.99, 1.01)	0.99	1.01 (1.00, 1.02)	0.09
Walking destination density (per 100)	1-mile	1.00 (0.97, 1.03)	0.85	**1.03 (1.00, 1.05)**	**0.03**
Network ratio	1-mile	1.04 (0.86, 1.25)	0.71	0.95 (0.81, 1.13)	0.57
Proportion retail	1-mile	1.58 (0.55, 4.44)	0.40	1.76 (0.70, 4.43)	0.23

Abbreviation: CI =Confidence Interval; CASI = Cognitive Abilities Screening Instrument; DSC = Digit Symbol Coding

[a] Continuous measures

[b] Maintained/improved score versus decline in score from Exam 5 to 6

[c] Controlling for age at Exam 5, sex, education, race/ethnicity, income, neighborhood socioeconomic status, site, APOE e4 carrier, neighborhood perception of safety walking day or night and crime, arthritis, cardiovascular and cerebrovascular disease, diabetes, number of residential moves

Table S6. Adjusted association between built environment characteristics and continuous cognitive change measures

Built environment characteristic measured at Exam 5 [a,b]	Variable name	Change in CASI score[c]		Change in DSC score[c]	
		Estimate (95% CI)	p-value	Estimate (95% CI)	p-value
Social destination density	SDD	-0.0020 (-0.0040, 0.0001)	0.06	0.0012 (-0.0038, 0.0062)	0.65
	SDD×years	0.0000 (-0.0002, 0.0003)	0.93	-0.0003 (-0.0009, 0.0003)	0.40
Walking destination density	WDD	-0061 (-0.0106, -0.0016)	0.009	0.0014 (-0.0098, 0.0126)	0.81
	WDD×years	-0.0001 (-0.0007, 0.0005)	0.70	-0.0009 (-0.0022, 0.0004)	0.19
Network ratio	NETRATIO	-1.33 (-3.20, 0.54)	0.17	-0.88 (-5.45, 3.67)	0.71
	NETRATIO×years	-0.01 (-0.33, 0.31)	0.96	-0.61 (-1.35, 0.12)	0.10
Proportion retail	PRET	-6.77 (-14.46, 0.85)	0.09	-1.75 (-20.12, 16.64)	0.85
	PRET×years	0.47 (-0.78, 1.72)	0.46	-1.41 (-4.24, 1.42)	0.33

Abbreviation: CI =Confidence Interval; CASI = Cognitive Abilities Screening Instrument; DSC = Digit Symbol Coding

[a] Measured in ½-mile radial buffer surrounding residence

[b] Continuous measures

[c] Controlling for age at Exam 5, sex, education, race/ethnicity, income, neighborhood socioeconomic status, site, APOE e4 carrier, neighborhood perception of safety walking day or night and crime, arthritis, cardiovascular and cerebrovascular disease, diabetes, number of residential moves

Table S7. Adjusted association between average annual built environment changes and maintained/improved cognition

		Maintained/improved CASI score[c,d]		Maintained/improved DSC score[c,d]	
Average annual change[a]	Buffer size	Log OR (95% CI)	p-value	Log OR (95% CI)	p-value
Social destination density	½-mile	1.0007 (0.9979, 1.0033)	0.63	1.0018 (0.9994, 1.0042)	0.14
Walking destination density	½-mile	1.0011 (0.9959, 1.0062)	0.69	1.0030 (0.9983, 1.0077)	0.22
Network ratio	½-mile	1.06 (0.14, 7.70)	0.95	0.64 (0.11, 3.86)	0.63
Proportion retail	½-mile	0.98 (0.00, 201.40)	0.99	0.96 (0.01, 111.90)	0.99
Social destination density	1-mile	1.0018 (0.9980, 1.0055)	0.36	1.0021 (0.9987, 1.0055)	0.23
Walking destination density	1-mile	1.0029 (0.9955, 1.0103)	0.45	1.0037 (0.9970, 1.0104)	0.28
Network ratio	1-mile	1.09 (0.11, 10.69)	0.94	0.58 (0.07, 4.53)	0.60
Proportion retail	1-mile	11.53 (0.02, 5612.40)	0.45	0.34 (0.00, 86.05)	0.71

Abbreviation: OR = odds ratio; CI =Confidence Interval; CASI = Cognitive Abilities Screening Instrument; DSC = Digit Symbol Coding

[a] Continuous measures

[b] Variable was log transformed and top and bottom 1 percentile deleted to improve efficiency of the estimate

[c] Maintained/improved score versus decline in score from Exam 5 to 6

[d] Controlling for age at Exam 5, sex, education, race/ethnicity, income, neighborhood socioeconomic status, site, APOE e4 carrier, neighborhood perception of safety walking day or night and crime, arthritis, cardiovascular and cerebrovascular disease, diabetes, number of residential moves
